# Methylglyoxal metabolism in trypanosomes and leishmania

**DOI:** 10.1016/j.semcdb.2011.02.001

**Published:** 2011-05

**Authors:** Susan Wyllie, Alan H. Fairlamb

**Affiliations:** Division of Biological Chemistry and Drug Discovery, Wellcome Trust Biocentre, College of Life Sciences, University of Dundee, Dow Street, Dundee, Angus DD1 5EH, Scotland, UK

**Keywords:** GLO1, glyoxalase I, GLO2, glyoxalase II, T[SH]_2_, trypanothione, *N*^1^,*N*^8^-bis(glutathionyl)spermidine, GSH, glutathione, LADH, lactaldehyde dehydrogenase, Trypanosoma, Leishmania, Methylglyoxal, Glyoxalase, Trypanothione, Drug discovery

## Abstract

Methylglyoxal is a toxic by-product of glycolysis and other metabolic pathways. In mammalian cells, the principal route for detoxification of this reactive metabolite is via the glutathione-dependent glyoxalase pathway forming d-lactate, involving lactoylglutathione lyase (GLO1; EC 4.4.1.5) and hydroxyacylglutathione hydrolase (GLO2; EC 3.2.1.6). In contrast, the equivalent enzymes in the trypanosomatid parasites *Trypanosoma cruzi* and *Leishmania* spp. show >200-fold selectivity for glutathionylspermidine and trypanothione over glutathione and are therefore sensu stricto lactoylglutathionylspermidine lyases (EC 4.4.1.-) and hydroxyacylglutathionylspermidine hydrolases (EC 3.2.1.-). The unique substrate specificity of the parasite glyoxalase enzymes can be directly attributed to their unusual active site architecture. The African trypanosome differs from these parasites in that it lacks GLO1 and converts methylglyoxal to l-lactate rather than d-lactate. Since *Trypanosoma brucei* is the most sensitive of the trypanosomatids to methylglyoxal toxicity, the absence of a complete and functional glyoxalase pathway in these parasites is perplexing. Alternative routes of methylglyoxal detoxification in *T. brucei* are discussed along with the potential of exploiting trypanosomatid glyoxalase enzymes as targets for anti-parasitic chemotherapy.

## 1 Introduction

Flagellated protozoa of the family Trypanosomatidae encompass a diverse range of organisms, including the human pathogens *Trypanosoma brucei*, *Trypanosoma cruzi* and *Leishmania* spp., causative agents of sleeping sickness, Chagas’ disease and leishmaniasis, respectively. These digenetic parasites undertake complex life cycles, differentiating into a variety of developmental forms while parasitizing both vertebrate and insect vector hosts. Collectively, the diseases are responsible for more than 120,000 fatalities annually and the loss of over 4,600,000 disability adjusted life years (DALYs) [1]. Some of the most socio-economically deprived regions of the world are afflicted by these vector-borne parasites and the accompanying economic burden provides a major obstacle to improving human health [2]. Almost all existing drugs used to treat these diseases suffer from serious problems ranging from severe toxic side effects [3] to acquired drug resistance [4,5]. To further compound these difficulties, treatments often require lengthy periods of hospitalisation and are prohibitively expensive [1]. Therefore, novel drug targets and more effective drug treatments are urgently required for these neglected diseases of poverty.

Metabolic pathways that are absent from, or significantly different to, host pathways are logical starting points for drug discovery. With this in mind, the glyoxalase pathway, a ubiquitous detoxification pathway that protects against the cellular damage caused by the toxic and mutagenic glycolytic metabolite methylglyoxal [6,7], would seem far from an ideal drug target within these parasites. The glyoxalase pathway comprises glyoxalase I (GLO1) (lactoylglutathione lyase, EC 4.4.1.5) and glyoxalase II (GLO2) (hydroxyacylglutathione hydrolase, EC 3.1.2.6), which act in concert to convert the spontaneously formed hemithioacetal adduct between glutathione and methylglyoxal into d-lactate and glutathione. The universal nature of the glyoxalase pathway emphasises its significance in general cellular function resulting in its conservation throughout evolution. However, quantitative differences in methylglyoxal metabolism of rapidly proliferating cells may be therapeutically exploitable. Elevated levels of GLOI, responsible for the initial step in the detoxification of methylglyoxal, have been found in tumour tissue from human colon, renal and prostate cancers [8] and are believed to be associated with the increased proliferative growth rates of tumours cells. Most significantly, inhibitors of GLO1 have been shown to be selectively toxic, not only to tumour cells [9], but also to other rapidly growing organisms such as the protozoan parasite *Plasmodium falciparum* [10]. These findings have raised the possibility that the glyoxalase pathway may indeed present a viable drug target in the Trypanosomatidae.

**Fig. 1:**
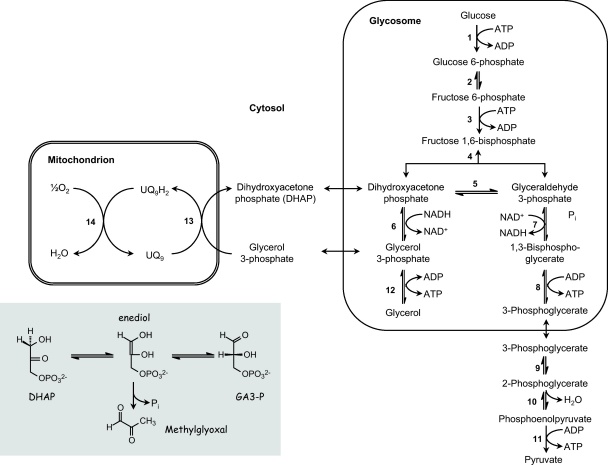
Glycolytic pathway and metabolic compartments in bloodstream form *T. brucei*. The shaded inset shows the route to methylglyoxal from dihydroxyacetone phosphate (DHAP) and glyceraldehyde 3-phosphate (GA-3P) via an enediol intermediate. Each mol of NADH generated in step 7 is reoxidised via the glycerophosphate shuttle (steps 6, 13 and 14) where glycerophosphate exits the glycosome in exchange for dihydroxyacetone phosphate. The location of enzymes in the glycosome and mitochondrion are bounded by single or double lines, respectively. Enzyme reaction steps are: 1, hexokinase; 2, glucose 6-phosphate isomerase; 3, phosphofructokinase; 4, aldolase; 5, triose phosphate isomerase; 6, glycerol 3-phosphate dehydrogenase (NAD+); 7, glyceraldehyde 3-phosphate dehydrogenase; 8, phosphoglycerate kinase; 9, phosphoglycerate mutase; 10, enolase; 11, pyruvate kinase; 12, glycerol kinase; 13, glycerol 3-phosphate dehydrogenase (FAD); 14; ubiquinol oxidase (trypanosome alternative oxidase). Other abbreviations: UQ_9_ and UQ_9_H_2_ are ubiquinone and ubiquinol, respectively.

The major source of methylglyoxal in cells is a by-product of glycolysis, where the triose phosphate intermediates dihydroxyacetone phosphate and glyceraldehyde 3-phosphate eliminate phosphate via an enediolate intermediate (Fig. 1, inset) [6]. Minor sources of methylglyoxal are from aminoacetone and hydroxyacetone, intermediates generated during catabolism of threonine and acetone [6]. Since *T. cruzi*, *T. brucei* and the *Leishmania* spp. are known to rapidly proliferate, demands for energy within cells are particularly high, resulting in high rates of glycolysis. Indeed, bloodstream-form *T. brucei* maintain respiratory rates approximately two orders of magnitude higher than those seen in mammalian cells [11]. Lacking cytochromes and a functional tricarboxylic acid cycle, this organism is entirely dependent on substrate-level phosphorylation from glycolysis for ATP production. The major end-product of the glycolytic pathway is pyruvate, rather than l-lactate, with net production of 2 mol of ATP per mol glucose consumed (Fig. 1). Lacking a canonical l-lactate dehydrogenase, NADH is oxidised by means of a plant-like glycerophosphate oxidase system that is not coupled to oxidative phosphorylation [12]. Glycolysis is unique in that the initial stages take place within a microbody-like organelle, the glycosome [13] and reducing equivalents from NADH are transferred to the mitochondrial glycerophosphate oxidase via the glycerophosphate/dihydroxyacetone phosphate shuttle. The insect stages of *T. brucei* and all stages of *T. cruzi* and *Leishmania* spp. also possess glycosomes, but are less dependent on glycolysis for ATP production (∼6–11-fold less than bloodstream forms [14]), because they have a full complement of cytochromes, partial tricarboxylic acid cycles and are able to use amino acids or lipids as alternative energy sources.

In the following review, studies which unravelled the unusual methylglyoxal metabolism of the trypanosomatids will be discussed, highlighting the remarkable difference between *T. brucei* that lacks a functional glyoxalase system and *T. cruzi* and *Leishmania* spp. that do. The potential chemotherapeutic value of this pathway will also be addressed.

## 2 Methylglyoxal detoxification in the trypanosomatids

### 2.1 Earliest observations

Until recently, relatively little was known about the glyoxalase pathway of the trypanosomatids. Early studies of glucose catabolism in *Leishmania braziliensis* revealed that these parasites released significant quantities of d-lactate [15]. Production of d-lactate in these cells was noted to increase markedly following incubation with glucose under anaerobic conditions. Since d-lactate is the end-product of methylglyoxal metabolism via the glyoxalase pathway, Darling and Blum went on to investigate the specific role of methylglyoxal in the production of d-lactate and found that *L. braziliensis* promastigotes, when incubated with methylglyoxal, secreted significant quantities of d-lactate [16]. These researchers were also able to detect GSH-dependent GLO1 and GLO2 activities in sonicated, undialysed lysates of these cells, confirming the presence of an active glyoxalase pathway apparently similar to other organisms [16]. Comparative studies of d- and l-lactate production in a further 4 strains of Leishmania and *T. lewisi* confirmed the production of d-lactate by parasites in the presence of glucose, most significantly under anaerobic conditions [17]. Intriguingly, in the same study with *T. brucei gambiense*, parasites failed to produce d-lactate when incubated with methylglyoxal, but excreted significant quantities of l-lactate instead. Since the canonical l-lactate dehydrogenase is absent from African trypanosomes, this provided the first indication that methylglyoxal detoxification mechanisms may not be identical across the Trypanosomatidae.

In subsequent studies by Ghoshal and colleagues, *Leishmania donovani* promastigotes were also shown to quantitatively convert methylglyoxal to d-lactate. However, in this instance, very limited GLO1 and GLO2 activities could be detected in dialysed extracts of these parasites using GSH as a co-factor [18]. The contrasting glyoxalase enzyme activities in dialysed versus undialysed lysates of *L. donovani* and *L. braziliensis* suggested that a factor essential to glyoxalase activity may have been lost during dialysis. These observations prompted our laboratory to investigate a role for the trypanosomatid thiol trypanothione in methylglyoxal detoxification. Trypanosomatid parasites are uniquely dependent upon trypanothione [T[SH]_2_, *N*^1^,*N*^8^-bis(glutathionyl) spermidine] [19] as their principal low molecular-mass thiol, which, together with trypanothione reductase, assumes many of the anti-oxidant functions commonly undertaken by glutathione [GSH] and glutathione reductase in their human hosts [20]. This dithiol is primarily responsible for the maintenance of thiol-redox homeostasis within trypanosomatids and is crucially involved in the protection of parasites from oxidative stress [21–23] and heavy metal toxicity [24,25]. The pivotal role T[SH]_2_ plays in several key metabolic pathways within these parasites prompted researchers to pose the question—could T[SH]_2_ play an equally important role in the detoxification of methylglyoxal in *Trypanosoma* and *Leishmania* spp.? Fifteen years following the initial studies of Darling and Blum, this lateral shift in thinking re-ignited research of methylglyoxal metabolism in the trypanosomatids [26,27].

### 2.2 Glyoxalase I

The metalloenzyme GLO1 isomerises glutathione hemithioacetal to S-d-lactoylglutathione, through proton transfer to a metal-bound enediol intermediate [28]. All previously characterized GLO1 enzymes, both prokaryotic and eukaryotic, utilise glutathione as a catalytic co-factor. Initial studies to determine whether T[SH]_2_ assumes this role in the trypanosomatids were carried out on the *Leishmania major* enzyme [26]. While recombinant GLO1 showed very little activity with glutathione hemithioacetal substrates, the parasite enzyme was found to be 200-fold more active with hemithioacetals formed with T[SH]_2_ and its metabolic precursor, *N*^1^-glutathionylspermidine. Moreover, this enzyme was also insensitive to glutathione derivatives that are potent inhibitors of all other characterized GLO1 enzymes. The unique substrate specificity demonstrated by *L. major* GLO1 in this study unequivocally defines this enzyme as T[SH]_2_-dependent and provided the first real evidence that the trypanosomatid glyoxalase pathway is unique among the eukaryotes.

**Fig. 2:**
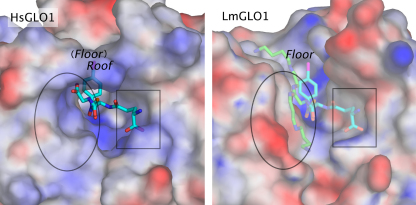
Comparison of the human and *L. major* GLO1 active sites. Density maps indicate the predicted and favourable interaction sites (XSITE) for binding of charged nitrogen (blue) and carboxylate oxygen (red) probes. A rectangle highlights the γ-glutamate-binding region; an ellipse, the glycyl carboxylate/amide-binding region. The cyan molecule is *S*-(*N*-hydroxy-*N-p*-iodophenylcarbamoyl)glutathione (from PDB 1QIN), a potent inhibitor of the human enzyme. This molecule is also shown modelled into the active site of the *L. major* enzyme (PDB2C1) alongside two possible binding conformations of the spermidine group of trypanothione and glutathionylspermidine, shown in green [31].

Unusual substrate specificity is not the only point of distinction between *L. major* GLO1 and other eukaryotic GLO1 enzymes. Characterisation of the recombinant *L. major* enzyme revealed a dependence upon nickel as a metal co-factor [26], in contrast to all other eukaryotic glyoxalase enzymes which utilise zinc [29]. This co-factor specificity squarely aligns *L. major* GLO1 with enzymes from bacteria such as *Escherichia coli*, *Pseudomonas aeruginosa* and *Yersinia pestis* [30]. Indeed, sequence analysis reveals that *L. major* GLO1 is most similar to bacterial glyoxalases, sharing 51% identity at the amino acid level to the equivalent enzyme from the cyanobacteria *Synechococcus* spp., while sharing only 33% identity with human GLO1 [26]. The differences in co-factor and substrate specificity in *L. major* GLO1 can be directly attributed to the unusual active site architecture, evident in the crystal structure of this enzyme [31]. In comparison to the human enzyme, *L. major* GLO1 maintains an increased negative charge and hydrophobic character within its active site which is believed to aid the accommodation of the positive/aliphatic glycyl-spermidine moiety of T[SH]_2_ or glutathionylspermidine (Fig. 2). In addition, a loop thought to be important in catalysis in human GLO1 is truncated within the parasite enzyme. Detailed three-dimensional analysis of human GLO1 in complex with a transition state analogue, identified three residues crucial in ligand binding [29]. Of these residues, Val149 and Lys150 in the human enzyme are replaced by acidic residues Asp100 and Tyr101 in both the *L. major* GLO1 and homologues subsequently identified in both *T. cruzi* [32] and *L. donovani* [33]. The conservation of this uncharged, aromatic and polar residue in the trypanosomatid GLO1 enzymes at this position suggests that this region of the active site may well be significant in determining substrate specificity for hemithioacetal derivatives with glutathionylspermidine or T[SH]_2_. Collectively, these structural studies confirm that there is considerable scope to target trypanosomatid GLO1 enzymes with specific inhibitors. The chemotherapeutic value of such inhibitors will be discussed in detail later in this review.

In subsequent years, GLO1 homologues from *L. donovani* and *T. cruzi* have been identified and characterized [32–34]. Metal reconstitution experiments showed that *T. cruzi* GLO1 was less fastidious than *L. major* GLO1 in its divalent metal ion requirement, with cobalt being as equally efficacious as nickel in restoring full enzyme activity [32]. As expected, GLO1 enzymes from these parasites closely mimicked the *L. major* enzyme in preferentially isomerising trypanothione hemithioacetals over those formed with glutathione (Table 1). Intriguingly, the *T. cruzi* enzyme showed a higher affinity for hemithioacetal adducts of glutathionylspermidine, as demonstrated by a markedly lower *K*_m_ value and a resulting 6-fold higher specificity constant, *k*_cat_/*K*_m_ [32]. In light of this observation, glutathionylspermidine adducts would appear to be the most efficient substrates for TcGLO1. However, it should be noted that trypanothione levels are 8-fold higher than glutathionylspermidine in *T. cruzi* epimastigotes [21]. Thus, it would seem that both glutathionylspermidine and T[SH]_2_ hemithioacetal adducts may act as the physiological substrates of the *T. cruzi* enzyme in vivo.

Our understanding of metabolic pathways in trypanosomatids has been greatly facilitated by the sequencing of representative genomes from the three major species: *T. brucei*, *T. cruzi* and *L. major* [35]. For the first time, this endeavour enabled a direct and global comparison of the metabolic pathways of all three pathogenic parasites [36]. One of the most intriguing discoveries arising from analysis of these genomes was the apparent absence of a gene encoding a GLO1 homologue in *T. brucei*. This has since been confirmed experimentally by the absence of detectable GLO1 activity in *T. brucei* whole cell extracts and the lack of d-lactate generation from whole cells incubated with methylglyoxal [34]. Despite having the highest glycolytic rate of all the trypanosomatids, bloodstream form *T. brucei* proved to be the least adept of the ‘Tritryp’ parasites in metabolising methylglyoxal, ultimately producing l-lactate rather than d-lactate as a metabolic end-product. The possibility that these parasites may use an alternative metabolic pathway will be discussed in detail in a subsequent section.

### 2.3 Glyoxalase II

The final step of methylglyoxal detoxification via the glyoxalase pathway is catalyzed by GLO2 which converts S-d-lactoylglutathione into d-lactate and free glutathione. At virtually the same time that GLO1 was defined as a T[SH]_2_-dependent enzyme in *L. major*, GLO2 from *T. brucei* was also shown to strongly prefer thioesters of T[SH]_2_ as substrates compared to those formed with glutathione (Table 2) [27]. Indeed, this study was closely followed by the identification of T[SH]_2_-dependent GLO2 enzymes in both *L. donovani* [37] and *T. cruzi* [34]. The characterization of GLO2 enzymes which depend upon T[SH]_2_ as a co-factor in both *Leishmania* spp. and *T. cruzi* confirms that these parasites maintain a complete, functional glyoxalase pathway that is significantly different to that of their vertebrate hosts. As in trypanosomatid GLO1 enzymes, the substrate specificity of GLO2 in these parasites can be directly related to specific amino acid substitutions within their active sites. Three basic residues that are known to play a crucial role in the binding of the glutathione moiety of GLO2 substrates in the human enzyme [38] are not conserved in the equivalent parasite enzymes [39]. It is assumed that these basic residues have been substituted in order to accommodate the positively charged thioesters of T[SH]_2_ or glutathionylspermidine. In addition, the crystal structure of the GLO2 from *L. infantum* has revealed the significance of strategically positioned polar residues within the active site responsible for binding the spermidine moiety of the thioester [39]. The mutually exclusive substrate specificities and significant differences in active site architecture between human and trypanosomatid GLO2 enzymes once again raise hopes that targeting of the parasite enzyme with specific inhibitors is an eminently achievable goal.

The retention of GLO2 in the absence of a functional GLO1 enzyme in *T. brucei* is somewhat perplexing but not entirely without precedence. Cestode and digenean parasitic helminthes have been studied that lack GLO1 while maintaining high levels of GLO2 activity [40]. Restoration of a functional glyoxalase system in *T. brucei* by expression of *T. cruzi* GLO1 resulted in increased resistance to methylglyoxal and increased conversion of methylglyoxal to d-lactate, demonstrating that GLO2 is functional in vivo [34]. However, it has been suggested that the true physiological function of GLO2 in the African trypanosome is unrelated to the detoxification of methylglyoxal [41]. Indeed, recombinant *T. brucei* GLO2 has demonstrated substrate promiscuity in hydrolyzing S-acetyl- and S-proprionyltrypanothione esters with the same catalytic efficiency as S-lactoyltrypanothione. The ease with which this enzyme hydrolyses a diverse range of thioesters has led to the hypothesis that GLO2 may function as a general T[SH]_2_-thioesterase in *T. brucei*.

### 2.4 Alternative metabolic pathways for the detoxification of methylglyoxal

Since methylglyoxal is generated primarily as a by-product of glycolysis, and the African trypanosome is solely dependent upon glycolysis for energy, it would be reasonable to assume that *T. brucei* would preserve robust methylglyoxal-metabolizing systems. Indeed several studies have shown these parasites to be particularly vulnerable to methylglyoxal toxicity [34,41], to dihydroxyacetone toxicity [42] and to a deficiency in triose phosphate isomerase activity [43]. In addition, it is noteworthy that the high glycolytic flux in bloodstream form *T. brucei* (9900 nmol h^−1^ [10^8^ cells]^−1^) [44] is associated with the production of l-lactate (30 nmol pyruvate produced at 37 °C h^−1^ [10^8^ cells]^−1^) [34]. Assuming this is generated exclusively from the glycolytic pathway this would represent 0.3% of the carbon flux. Although somewhat higher than that of mammalian erythrocytes (0.089%), this correlates with the higher steady state concentrations of dihydroxyacetone phosphate and glyceraldehyde 3-phosphate in bloodstream forms (117 and 4 μM versus 1000 and 69 μM, respectively for erythrocytes [45] and trypanosomes [46]) (recalculated assuming 1 g wet weight is 1.7 × 10^10^ cells and 1 × 10^8^ cells = 5.8 μl). These observations pose the question—in the absence of a functional glyoxalase system how do these parasites protect themselves from this toxic metabolite?

In a previous study, Ghoshal and co-workers identified NADPH-dependent methylglyoxal reductase activity in *L. donovani* promastigotes [18]. These parasites were shown to metabolize approximately 1% of the exogenous methylglyoxal added to cultures via this reductase, generating l-lactaldehyde as an end-product. In view of the generation of high levels of l-lactate by methylglyoxal-treated *T. brucei*, our laboratory proposed that methylglyoxal reductase activity may be elevated in *T. brucei* to compensate for the absence of GLO1. Indeed, when NADPH-dependent methylglyoxal reductase activity was measured in all three trypanosomatid cell lysates, a two-fold higher reductase activity was observed in *T. brucei* procyclic and bloodstream extracts, compared to that seen in *L. major* and *T. cruzi* cells. Bearing in mind the rates of methylglyoxal metabolism measured in these cells, elevated methylglyoxal reductase activity could conceivably account for all methylglyoxal metabolism measured in *T. brucei*. To date, a definitive methylglyoxal reductase gene has yet to be identified in *T. brucei*. However, two putative aldo-keto-reductase genes (Tb927.2.5180 and Tb11.02.3040) which are members of the same aldo-keto reductase superfamily as methylglyoxal reductase, have been annotated in the genome although not functionally characterized. Recent studies have identified a close homologue of these enzymes in *L. donovani* [47]. While this recombinant aldose reductase was found to effectively reduced methylglyoxal substrates, it somewhat surprisingly showed a 4-fold preference for glutathione as a co-factor rather than T[SH]_2_. Clearly, further studies will be required to define both the functionality and thiol-preference of the putative aldo-keto-reductases from *T. brucei*.

It is important to note that this hypothesis is not universally accepted. In a study carried by Krauth-Siegel and co-workers [41], methylglyoxal reductase activities in bloodstream *T. brucei* extracts were found to be 50-fold lower than those detected in our own study and between 20 and 40 times lower than those measured in different *Leishmania* spp. There are no obvious reasons for the disparate methylglyoxal reductase activities measured in the two studies, however, it is clear that reductase activities at the lower level would be unable sustain methylglyoxal metabolism in *T. brucei*.

To complete the metabolism of methylglyoxal reductase-generated l-lactaldehyde to l-lactate, *T. brucei* would require a functional l-lactaldehyde dehydrogenase. While lactaldehyde dehydrogenase activity was detected in procyclic *T. brucei* cell lysates, a similar activity could not be detected in lysates of the bloodstream stage of the parasite [34]. Failure to detect NAD^+^-dependent lactaldehyde dehydrogenase activities has been attributed to a high endogenous rate of NADH oxidation in bloodstream lysates masking the formation of NADH. Nevertheless, these studies confirm that procyclic *T. brucei* are capable of metabolizing methylglyoxal to l-lactate. It remains to be seen whether this is also true of the bloodstream trypanosome.

In addition to the glyoxalase system and methylglyoxal reductase, two alternative routes of methylglyoxal detoxification have been described in mammalian cells. These mechanisms of detoxification are dependent on methylglyoxal dehydrogenase enzymes, 2-oxaldehyde dehydrogenase and betaine aldehyde dehydrogenase [48]. Both enzymes catalyze the oxidation of methylglyoxal to pyruvate. However, no homologues of these enzymes have been identified in the *T. brucei* genome and neither NAD^+^ nor NADP^+^-dependent methylglyoxal dehydrogenase activities were detected in *T. brucei* cell extracts. Thus, the exact mechanisms of methylglyoxal detoxification in the African trypanosome remain an enigma.

## 3 Prospects for parasite chemotherapy

**Fig. 3:**
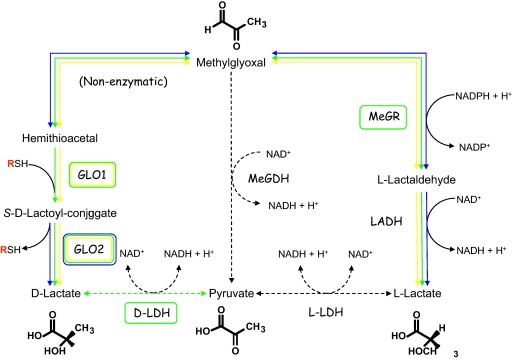
Metabolism of methylglyoxal in trypanosomatids. In *T. cruzi* and *L. major*, the principal end-product of methylglyoxal metabolism is d-lactate. In the absence of GLO1, *T. brucei* does not possess a functional GLO system and may metabolize methylglyoxal via methylglyoxal reductase (MeGR) and lactaldehyde dehydrogenase (LADH) to l-lactate. Solid lines: confirmed metabolic steps in the trypanosomatids; dashed lines: absent or unconfirmed metabolic steps. *T. cruzi*, *L. major* and *T. brucei* shown in yellow, green and blue, respectively. Other abbreviations: MeGDH, methylglyoxal dehydrogenase; LDH, lactate dehydrogenase.

The unique substrate specificities and unusual active site architecture of trypanosomatid glyoxalase enzymes suggests that they may be exploitable as targets for anti-parasitic chemotherapy (Fig. 3). Without question these enzymes can be selectively targeted by inhibitors based upon their substrate specificity, as demonstrated by the potent and specific inhibition of *L. major* GLO1 with S-4-bromobenzyl glutathionylspermidine and S-4-dinitrophenylglutathionylspermidine [31,34]. Indeed, the generation of high resolution crystal structures of both the *L. major* GLO1 [31] and *L. infantum* GLO2 [39] has raised hopes that the design of more potent and specific inhibitors of these enzymes is eminently achievable. However, quantitative assessment of the glyoxalase pathway of *L. infantum* by modeling and computer simulation has recently brought into question the whole validity of this metabolic pathway as a therapeutic target in these parasites [49]. Using experimentally determined kinetic parameters and metabolite concentrations, the importance of both GLO1 and GLO2 in maintaining a low intracellular methylglyoxal concentration within these cells was estimated to be relatively low. In fact, the rate of methylglyoxal production in cells along with intracellular T[SH]_2_ concentrations were identified as the critical parameters in controlling levels of methylglyoxal. With the rationale that the success of this metabolic pathway as a viable drug target relies upon increasing methylglyoxal concentrations in cells, this simulated study appears to nullify the chemotherapeutic potential of inhibitors targeted against both glyoxalase enzymes in *L. infantum*. These findings are in direct contradiction to recent studies of GLO1 gene deletion mutants of *L. donovani* [50]. Here, Chauhan and Madhubala demonstrated that removal of a single copy of the GLO1 gene by targeted gene replacement resulted in a markedly slower growth rate in promastigotes. They went on to show that deletion of the second genomic copy could only be achieved in the presence of an episomal “rescue” copy of GLO1, a behaviour that is generally accepted as indicative of essentiality in *Leishmania* spp. [51,52]. Taken together, these observations support the assertion that GLO1 plays a significant and essential role in survival of *L. donovani* promastigotes and suggest that potent inhibitors targeting this enzyme are likely to prove lethal to this pathogenic organism.

In comparison to GLO1, GLO2 may be a less promising target for chemotherapy. Since the reaction catalyzed by GLO1 is essentially irreversible under physiological conditions, the inhibition of GLO2 would merely lead to the accumulation of the non-toxic metabolite S-d-lactoyltrypanothione, which, in turn, would be predicted to be able to displace competitive inhibitors from GLO2. However, this would be associated with a depletion of glutathionylspermidine and T[SH]_2_ cellular content that could disrupt the other essential metabolic functions of these unique metabolites. To date, the only genetic studies of GLO2 essentiality in the trypanosomatids have been in the African trypanosome where RNAi and gene deletion studies in the procyclic form of the parasite demonstrated the enzyme to be non-essential [41,41]. Since GLO2 is not believed to play a significant role in methylglyoxal detoxification in *T. brucei*, these findings have little or no bearing on the essentiality of GLO2 enzymes in the other trypanosomatids. Further genetic and biochemical studies are required to establish the therapeutic value of inhibitors which target parasitic GLO2 enzymes.

## 4 Concluding remarks

Our understanding of the glyoxalase pathway in trypanosomatid parasites has greatly improved in the last 5–10 years. Comprehensive biochemical and molecular studies of this pathway, facilitated by the sequencing of the “TriTryp” genomes, has highlighted significant differences in the way these parasites metabolize methylglyoxal. Several studies have suggested that the contrasting substrate specificities of the human and trypanosomatid glyoxalase enzymes (GLO1 and GLO2), discussed at length in this review, make them attractive targets for rational drug design. While this may well be the case in *T. cruzi* and *Leishmania* spp., it is unlikely to be true in the African trypanosome which lacks a functional glyoxalase pathway. Identifying the principal routes of methylglyoxal detoxification in *T. brucei* is likely to be a focus of future studies in this field. It is hoped that a full understanding of the “unusual” methylglyoxal metabolism of *T. brucei* may lead to the discovery of additional drug targets within these parasites.

## Figures and Tables

**Table 1:** Kinetic parameters of human and trypanosomatid GLO1 enzymes.

GLO1	Methylglyoxal hemithioacetal substrate	*K*_m_, μM	*k*_cat_, s^−1^	*k*_cat_/*K*_m_, ×10^7^ M^−1^ s^−1^	Relative, *k*_cat_/*K*_m_
*L. major*[31]	T[SH]_2_	32 ± 3	800 ± 30	1.5	60
	GspdSH	71 ± 5	1070 ± 40	2.5	100
	GSH	>1900	ND	0.009	0.36

*T. cruzi*[32]	T[SH]_2_	109 ± 10	363 ± 33	3.3	16.5
	GspdSH	8.0 ± 0.4	161 ± 12	20	100
	GSH	>1800	ND	0.0014	0.007

Human [31]	T[SH]_2_	130 ± 12	104 ± 6	0.08	2.9
	GspdSH	148 ± 9	83 ± 4	0.06	2.1
	GSH	49 ± 3	1360 ± 40	2.8	100

**Table 2:** Kinetic parameters of mammalian and trypanosomatid GLO2 enzymes.

GLO2	Lactoyl-thiol substrate	*K*_m_, μM	*k*_cat_, s^−1^^a^	*k*_cat_/*K*_m_, ×10^5^ M^−1^ s^−1^
*L. donovani*[37]	T[SH]_2_	39	ND	ND
	GSH	ND	ND	ND

*T. brucei*[27]	T[SH]_2_	164 ± 18	49.2	3.0
	GSH	>3000	>4.5	0.015

Mammalian [53]	T[SH]_2_	ND	ND	ND
	GSH	190 ± 1	4.37	0.23

a Values calculated from published data.
